# Global dynamics of a novel deterministic and stochastic SIR epidemic model with vertical transmission and media coverage

**DOI:** 10.1186/s13662-020-03145-3

**Published:** 2020-12-04

**Authors:** Xiaodong Wang, Chunxia Wang, Kai Wang

**Affiliations:** Department of Medical Engineering and Technology, Xinjiang Medical University, Urumqi, Xinjiang 830011 P.R. China

**Keywords:** SIR epidemic model, Extinction, Persistence in mean, Vertical transmission, Media coverage

## Abstract

In this paper, we study a novel deterministic and stochastic SIR epidemic model with vertical transmission and media coverage. For the deterministic model, we give the basic reproduction number $R_{0}$ which determines the extinction or prevalence of the disease. In addition, for the stochastic model, we prove existence and uniqueness of the positive solution, and extinction and persistence in mean. Furthermore, we give numerical simulations to verify our results.

## Introduction

To the best of our knowledge, vaccination is one of the most effective ways to treat and prevent diseases. It has been used to restrain diseases such as tetanus, diphtheria, rubella, mumps, pertussis, measles, hepatitis B and influenza [1]. For instance, during the outbreak of SARS in 2003 [2], H1N1 influenza pandemic in 2009 [3], and H7N9 influenza in 2013 [4], unprecedented mass influenza vaccination programs were launched by a large number of countries to timely immunize as many people as possible. Those strategies greatly controlled the spread of infection and then decreased the incidence rate [5]. In addition, with the development of information technology, media reports play an important role in the prevention and control of diseases, for example, during the outbreak of SARS and H1N1, media reports effectively stopped the spread of the disease and provided scientific and reasonable preventive measures for people [6–9]. However, in order to use media with high efficiency to control diseases, it is necessary to describe the quantitative relationship between the number of infections and media coverage with mathematical formula. Recently, many scholars researchers have carried out wide studies and obtained a great deal of achievement in this field (see [10, 11]). Most of them assumed that when there is no infection there is no media coverage of infectious diseases, the more infected individuals, the more media coverage. Liu et al. [12] established an SEIH epidemic model with incidence rate $\beta {e^{(-a_{1}E-a_{2}I-a_{3}H)}SI}$ and found that media coverage is not a key factor in determining whether or not a disease will break out, but it has a evident impact on the scale of the spread of disease. Cui et al. [13] presented an SEI epidemic model with incidence rate $\beta {e^{-mI}SI}$ and found the disease can be controlled when the media impact is stronger. Tchuenche et al. [14] discuss how media coverage has impact on the disease by constructing a new constant rate $(\beta _{1}-\frac{\beta _{2}I}{\eta +I})$, where $\beta _{1}$ is the usual valid contact rate, $\beta _{2}$ is the maximum reduced valid contact rate through actual media coverage, and $\eta (\eta >0)$ is the rate of the reflection on the disease. On the other hand, media coverage cannot completely prevent disease transmission, so we have $\beta _{1}>\beta _{2}$. Moreover, other forms, such as $(\mu _{1}-\mu _{2}f(I))\frac{SI}{S+I}$, $\beta e^{-\epsilon m M}$, $\beta e^{-\alpha I(t-\tau )}$, have been proposed to describe the media-induced incidence rate (see [15–17]). In addition to media reports, vertical transmission can also affect the spread of diseases; in vertical transmission, the offspring of infected parents may already be infected with the disease at birth [18–20], such as rubella, herpes simplex, hepatitis B, Chagas’ disease and AIDS. Meng and Chen [21] proposed a new SIR epidemic model with vertical and horizontal transmission, they compared the validity of the strategy of pulse vaccination with no vaccination and constant vaccination, and concluded that a pulse vaccination strategy is more effective than no vaccination and continuous vaccination. In [22], they considered a non-linear mathematical model for HIV epidemic that spreads in a variable size population through both horizontal and vertical transmission and found that by controlling vertical transmission rate, the spread of the disease can be significantly reduced; the equilibrium values of infective and AIDS population can be maintained at the desired levels.

**Figure 1:**
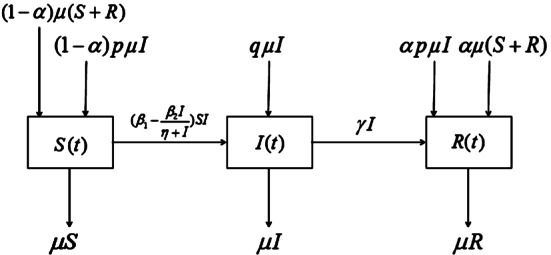
The compartmental diagram and model equation for a novel SIR epidemic model with vertical transmission and media coverage

Motivated by the above work, in this paper, we build a new SIR epidemic model with both vertical transmission and media coverage and give a compartmental diagram (see Fig. 1) as follows:

1.1 $$ \textstyle\begin{cases} {\frac{dS}{dt} =-(\beta _{1}-\frac{\beta _{2}I}{\eta +I}){SI}-\mu {S}+(1- \alpha ){p\mu {I}}+(1-\alpha ){\mu (S+R)},} \\ {\frac{dI}{dt} = (\beta _{1}-\frac{\beta _{2}I}{\eta +I}){SI}-\mu {I}- \gamma {I}+q\mu {I},} \\ {\frac{dR}{dt} =\gamma {I}-\mu {R}+\alpha {p\mu {I}}+\alpha {\mu (S+R)}.} \end{cases} $$

The parameters in the model (1.1) are summarized in the following list:

- $\beta _{1}$: the usual valid contact rate.
- $\beta _{2}$: the maximum reduced valid contact rate through actual media coverage.
- *η*: the rate of the reflection on the disease.
- *μ*: who are born and die at the same rate.
- *γ*: the recovery rate of the infected individuals.
- *p*: the proportion of the offspring of infective parents that are susceptible individuals.
- *q*: the proportion of the offspring of infective parents that are infective individuals.
- *α*: the proportion of those vaccinated successfully to the entire susceptible including mature species.

Here the constants $0 < p < 1,0 < q < 1, p+q=1$, $0<\alpha <1$ and the other parameters are nonnegative.

In addition, one can see that the population has a constant size, which is normalized to unity

$$ S(t)+I(t)+R(t)=1. $$

Hence, we only need to consider the SI model as follows:

1.2 $$ \textstyle\begin{cases} {\frac{dS}{dt} =-(\beta _{1}-\frac{\beta _{2}I}{\eta +I}){SI}-\mu {S}-(1- \alpha )\mu {q}I+(1-\alpha ){\mu },} \\ {\frac{dI}{dt} = (\beta _{1}-\frac{\beta _{2}I}{\eta +I}){SI}-(p\mu + \gamma )I.} \end{cases} $$

Clearly, $\Gamma =\{(S,I)|S,I\geq 0,S+I<1\}$ is an invariant set of the model (1.2).

On the other hand, one neglected the effect of the environment noise for the disease in model (1.2), in fact, in the process of transmission, the disease inevitably was affected by environmental noise (see e.g. [23, 24]). Therefore, deterministic epidemic models cannot accurately predict the future dynamics of infectious diseases, while stochastic models can make and many stochastic models for an epidemic have been built (see e.g. [25–27]). In [28], Ji et al. discussed a stochastic SIR model and found the disease shows persistence under some conditions. In [29], Yang et al. studied the global threshold dynamics for a stochastic SIS epidemic model incorporating media coverage and gave the basic reproduction number which determines the persistence or extinction of the disease.

Next we introduce stochastic perturbations using a method similar to that in [30] of the model (1.2) and the model equation as follows:

1.3 $$ \textstyle\begin{cases} dS=[-(\beta _{1}-\frac{\beta _{2}I}{\eta +I}){SI}-\mu {S}-(1-\alpha ) \mu {q}I+(1-\alpha ){\mu }]\,dt-\sigma SI\,dB(t), \\ {dI= [(\beta _{1}-\frac{\beta _{2}I}{\eta +I}){SI}-(p\mu +\gamma )I]\,dt+ \sigma SI\,dB(t),} \end{cases} $$

where $B(t)$ is a one-dimensional standard brownian motion on some probability space, and *σ* is the intensity of $B(t)$.

The rest of this paper is organized as follows. In Sect. 2, we study that the dynamic behavior of the deterministic model (1.2). In Sect. 3, we discuss the dynamic behaviors of the stochastic model (1.3) including the extinction and persistence in mean. In Sect. 4, we present numerical simulations to verify our results. In Sect. 5, we give a brief summary of our results. In the Appendix, we will give some proofs of the main results.

## The dynamic behaviors of the deterministic model (1.2)

### Equilibria and stability

Clearly, the model (1.2) has two equilibria, that is, the first one is the disease-free equilibrium $E_{0}=(S_{0},0)$, where $S_{0}=1-\alpha $. The second one is the endemic equilibrium $E_{1}=(S^{*},I^{*})$ which satisfies

2.1 $$\begin{aligned} \begin{aligned} & \biggl(\beta _{1}-\frac{\beta _{2}I^{*}}{\eta +I^{*}} \biggr){S^{*}I^{*}}-(p\mu + \gamma )I^{*}=0, \\ &{-} \biggl(\beta _{1}-\frac{\beta _{2}I^{*}}{\eta +I^{*}} \biggr){S^{*}I^{*}}- \mu {S^{*}}-(1- \alpha )\mu {q}I^{*}+(1-\alpha ){\mu }=0. \end{aligned} \end{aligned}$$

From the first equation of (2.1), we get

2.2 $$ S^{*}= \frac{p\mu +\gamma }{\beta _{1}-\frac{\beta _{2}I^{*}}{\eta +I^{*}}}. $$

Substituting (2.2) into the second equation of (2.1), we get $f(I)=g(I)$, where

$$\begin{aligned} &f(I)=(1-\alpha )\mu - \bigl[(1-\alpha )\mu {q}+p\mu +\gamma \bigr]I, \\ &g(I)= \frac{\mu (p\mu +\gamma )}{(\beta _{1}-\frac{\beta _{2}I}{\eta +I})}. \end{aligned}$$

By calculating the derivative of the function $g(I)$, we have

$$ g'(I)= \frac{\beta _{2}\eta \mu (p\mu +\gamma )}{[\beta _{1}\eta +(\beta _{1}-\beta _{2})I]^{2}}>0, $$

so we see that $g(I)$ is monotone increasing with respect to *I*. Similarly, $f'(I)=-[(1-\alpha )\mu {q}+p\mu +\gamma ]<0$, which indicates $f(I)$ is monotonous decreasing with respect to *I*. When $I=1$, we can get $f(1)<0<g(1)$, when $I=0$, $f(0)=\mu (1-\alpha ),g(0)=\frac{\mu (p\mu +\gamma )}{\beta _{1}}$.

If $f(0)=\mu (1-\alpha )< g(0)=\frac{\mu (p\mu +\gamma )}{\beta _{1}}$, namely, $\frac{\beta _{1}(1-\alpha )}{p\mu +\gamma }=R_{0}<1$, $f(I)$ and $g(I)$ non-intersect, that is to say, model (1.2) has no endemic equilibrium if $R_{0}<1$.

If $f(0)=\mu (1-\alpha )>g(0)=\frac{\mu (p\mu +\gamma )}{\beta _{1}}$, that is, $\frac{\beta _{1}(1-\alpha )}{p\mu +\gamma }=R_{0}>1$, there exists $I^{*}\in (0,1)$ such that $f(I^{*})=g(I^{*})$, in other words, model (1.2) has a unique endemic equilibrium if $R_{0}>1$. Here $R_{0}=\frac{\beta _{1}(1-\alpha )}{p\mu +\gamma }$ is the basic reproduction number of model (1.2).

#### Theorem 2.1

*The disease*-*free equilibrium* $E_{0}$ *of model* (1.2) *is globally asymptotically stable if* $R_{0}<1$, *the endemic equilibrium* $E_{1}$ *is globally asymptotically stable if* $R_{0}>1$.

## The dynamic behavior of the stochastic model (1.3)

### Preliminaries

Throughout this paper, we let $(\Omega, \{\mathcal{F}\}_{t\geq 0}, P)$ be a complete probability space with a filtration $\{\mathcal{F}\}_{t\geq 0}$ satisfying the usual conditions (that is to say, it is increasing and right continuous while $\mathcal{F}_{0}$ contains all *P*-null sets). Denote $\mathbb{R}_{+}^{d}=\{x\in \mathbb{R}^{d}|x_{i}>0,0\leq i\leq d\}$.

### Existence and uniqueness of positive solution

#### Theorem 3.1

*There is a unique solution* $(S (t ),I (t ) )$ *of model* (1.3) *on* $t\geq 0$ *for any initial value* $(S(0),I(0))\in \mathbb{R}_{+}^{2}$, *and the solution will remain in* $\mathbb{R}_{+}^{2}$ *with probability one*, *namely*, $(S (t ),I (t ) ) \in \mathbb{R}_{+}^{2}$ *for all* $t\geq 0$ *almost surely*.

### Extinction

In this section, we will give the condition of the disease to die out; firstly, we show there is a unique global and positive solution of model (1.3). For convenience, we define $\langle X(t)\rangle =\frac{1}{t}\int _{0}^{t}X(s)\,ds$.

#### Theorem 3.2

*For any initial value* $(S(0),I(0))\in \mathbb{R}_{+}^{2}$, *if* $\sigma ^{2}>\frac{\beta _{1}^{2}}{2(p\mu +\gamma )}$ *or* $\sigma ^{2}\leq \beta _{1}$ *and* $\beta _{1}< p\mu +\gamma +\frac{\sigma ^{2}}{2}$ *holds*, *then the disease* $I(t)$ *will die out exponentially with probability one*; *furthermore*,

$$ \lim_{t\rightarrow \infty } \bigl\langle S(t) \bigr\rangle =1- \alpha,\quad \textit{a.s.} $$

### Persistence in mean

In section, we will discuss the persistence of the disease $I(t)$.

#### Theorem 3.3

*Let* $(S (t ), I (t ) )$ *be the solution of system* (1.3) *with any initial value* $(S (0 ), I (0 ) )\in \mathbb{R}_{+}^{2}$, *if* $\sigma ^{2}<\min \{(\beta _{1}-\beta _{2})(1-\alpha ), \frac{2(p\mu +\gamma )(R_{0}-1)}{(1-\alpha )^{2}}, \frac{\beta _{1}}{1-\alpha }\}$ *and* $R_{0}>1$, *then the solution* $(S (t ), I (t ) )$ *of the proposed model* (1.3) *has the following property*:

$$ I_{2}\leq \liminf_{t\rightarrow +\infty } \bigl\langle I(t) \bigr\rangle \leq \limsup_{t\rightarrow +\infty } \bigl\langle I(t) \bigr\rangle \leq I_{1}\quad \textit{a.s.}, $$

*where*

$$ I_{1}= \frac{\mu [\beta _{1}(1-\alpha )-(p\mu +\gamma +\frac{\sigma ^{2}}{2}(1-\alpha )^{2})]}{[\mu (1-\alpha q)+\gamma ][\beta _{1}-\sigma ^{2}(1-\alpha )]} \quad \textit{and} \quad I_{2}= \frac{\mu ((\beta _{1}-\beta _{2})(1-\alpha )-\sigma ^{2})}{2(\beta _{1}-\beta _{2})[\mu (1-\alpha q)+\gamma ]}. $$

## Numerical analysis

In this section, we use hepatitis B as an example. We use the Runge–Kutta method to find the numerical simulation of the ODE model (1.2) and the stochastic epidemic model (1.3). This verifies our analytical results. To demonstrate the influence of the stochastic process, we perform simulations for the stochastic model and its corresponding deterministic model version.

**Figure 2:**
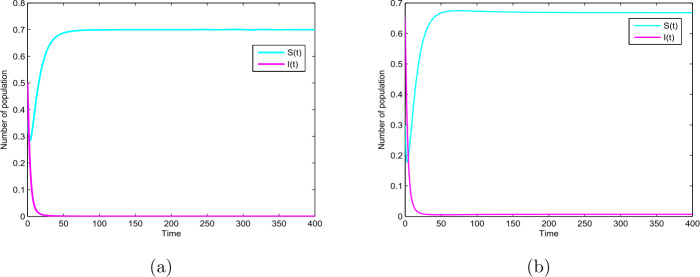
The paths $S(t)$ and $I(t)$ for the model (1.2) with $R_{0}=0.91<1$ and $R_{0}=1.05>1$

Firstly, we choose $p=0.6$, $q=0.4$ and other parameter values given by Table 1. In this case, the basic reproduction number of the ODE model (1.2) $R_{0}=0.91<1$, then the ODE model (1.2) have a disease-free equilibrium which is globally asymptotically stable (see Theorem 2.1), as shown in Fig. 2(a).

**Table 1:** Parameters of the model (1.2)

Symbol	Value	References
*μ*	0.1	[31]
$\beta _{1}$	0.6	[31]
$\beta _{2}$	0.1	[32]
*γ*	0.4	[32]
*α*	0.3	[33]
*η*	10	[32]
p(p+q=1)	[0.6,0.01,0.1]	Assumed
q	[0.4,0.99,0.9]	Assumed

Secondly, we choose $p=0.01$, $q=0.99$ and other parameter values given by Table 1. In this case, the basic reproduction number of the ODE model (1.2) $R_{0}=1.05>1$, then the ODE model (1.2) has an endemic equilibrium which is globally asymptotically stable (see Theorem 2.1), as shown in Fig. 2(b).

**Figure 3:**
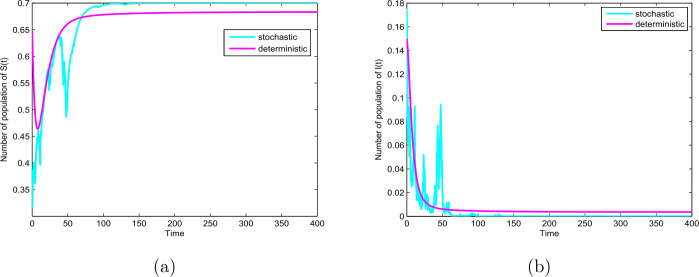
The path $S(t)$ and $I(t)$ for the model (1.2) and (1.3) with $R_{0}=1.02>1$ and $0.64=\sigma ^{2}>\frac{\beta _{1}^{2}}{2(p \mu +\gamma )}=0.44$

**Figure 4:**
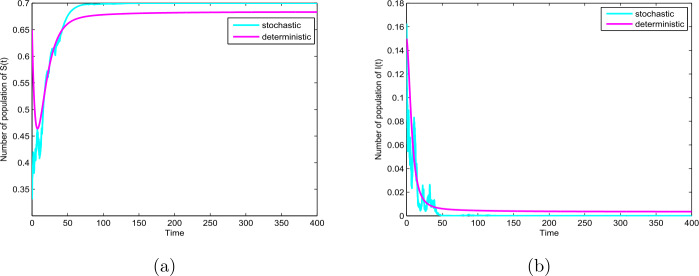
The paths $S(t)$ and $I(t)$ for the model (1.2) and (1.3) with $R_{0}=1.02>1$ and $0.49=\sigma ^{2}<0.6=\beta _{1}$ and $0.6=\beta _{1}< p \mu +\gamma +0.5\sigma ^{2}=0.66$

Thirdly, we choose $p=0.1$, $q=0.9$, $\sigma =0.8$ and the other parameter values given by Table 1. In this case, we have $0.64=\sigma ^{2}>\frac{\beta _{1}^{2}}{2(p \mu +\gamma )}=0.44$, then the disease will die out (see Theorem 3.2 and Fig. 3(b)). In addition, let $\sigma =0.7$ and take unchanged other parameters, we have $0.49=\sigma ^{2}<0.6=\beta _{1}$ and $0.6=\beta _{1}< p \mu +\gamma +0.5\sigma ^{2}=0.66$, similarly, then the disease will die out (see Theorem 3.2 and Fig. 4(b)). On the other hand, the basic reproduction number of the ODE model $R_{0} =1.02>1$, this means that the ODE model (1.2) also has an endemic equilibrium which is globally asymptotically stable, as shown in Fig. 3 and Fig. 4. Our results reveal that random perturbations in the environment can restrain the spread of the disease.

Finally, we choose $\beta _{1}=0.9$, $\beta _{2}=0.5$, $p=0.1$, $q=0.9$, $\sigma =0.4$ and other parameter values given by Table 1. In this case, we have

$$\begin{aligned} &R_{0}=1.54>1, \\ &0.16=\sigma ^{2}< \min \biggl\{ (\beta _{1}-\beta _{2}) (1-\alpha ), \frac{2(p\mu +\gamma )(R_{0}-1)}{(1-\alpha )^{2}}, \frac{\beta _{1}}{1-\alpha } \biggr\} \\ &\phantom{0.16}=\min \{0.28,0.90,1.29\}=0.28, \\ &I_{1}=\frac{\mu [\beta _{1}(1-\alpha )-(p\mu +\gamma +\frac{\sigma ^{2}}{2}(1-\alpha )^{2})]}{[\mu (1-\alpha q)+\gamma ][\beta _{1}-\sigma ^{2}(1-\alpha )]}=0.0485, \\ &I_{2}=\frac{\mu ((\beta _{1}-\beta _{2})(1-\alpha )-\sigma ^{2})}{2(\beta _{1}-\beta _{2})[\mu (1-\alpha q)+\gamma ]}=0.0317. \end{aligned}$$

**Figure 5:**
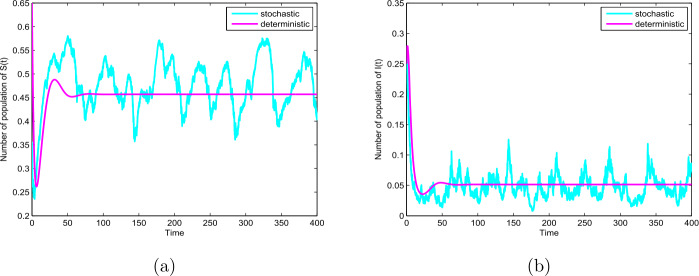
The paths $S(t)$ and $I(t)$ for the model (1.2) and (1.3) with $R_{0}=1.54>1$ and $0.16=\sigma ^{2}<\min \{(\beta _{1}-\beta _{2})(1-\alpha ), \frac{2(p\mu +\gamma )(R_{0}-1)}{(1-\alpha )^{2}}, \frac{\beta _{1}}{1-\alpha }\} =\min \{0.28,0.90,1.29\}=0.28$

Then the disease $I(t)$ will show persistence in mean, namely, the disease will prevail (see Theorem 3.3 and Fig. 5(b)).

## Conclusion

In this paper, we study a novel deterministic and stochastic SIR epidemic model with vertical transmission and media coverage. For the deterministic model (1.2), we define a threshold parameter $R_{0}=\frac{\beta _{1}(1-\alpha )}{p\mu +\gamma }$ which completely determines extinction and prevalence of the disease. Our results show that the disease-free equilibrium $E_{0}$ for model (1.2) is globally asymptotically stable if $R_{0}<1$, the endemic equilibrium $E_{1}$ is globally asymptotically stable if $R_{0}>1$ (see Theorem 2.1 and Fig. 2(a)–(b)). In addition, for the corresponding stochastic model (1.3), we obtain the sufficient condition of the extinction of the disease, namely, if $\sigma ^{2}>\frac{\beta _{1}^{2}}{2(p\mu +\gamma )}$ or $\sigma ^{2}\leq \beta _{1}$ and $\beta _{1}< p\mu +\gamma +\frac{\sigma ^{2}}{2}$ hold, then the disease $I(t)$ will exponentially die out with probability one (see Theorem 3.2 and Fig. 3 and Fig. 4). Furthermore, $\lim_{t\rightarrow \infty }\langle S(t)\rangle =1-\alpha$, a.s. (see Theorem 3.2). By Theorem 3.2 we can find that when Theorem 3.2 holds, the disease will die out, but for the corresponding deterministic model (1.2), $R_{0}>1$, there exists an endemic equilibrium $E_{1}$, which means that a stochastic perturbation can restrain the outbreak of the disease (see Fig. 3 and Fig. 4). Furthermore, from Theorem 3.3, if $\sigma ^{2}<\min \{(\beta _{1}-\beta _{2})(1-\alpha ), \frac{2(p\mu +\gamma )(R_{0}-1)}{(1-\alpha )^{2}}, \frac{\beta _{1}}{1-\alpha }\}$ and $R_{0}>1$, then the disease is persistent in mean (see Fig. 5).

Some topics deserve further study. For example, one may construct some more realistic but complex models, such as considering the effects of delay, complex network, pulse vaccination and Lévy noise. Some scholars have already done a great deal of work (see [34–40]). We leave these investigations for future work.

## Data Availability

Data sharing not applicable to this article as no datasets were generated or analyzed during the current study.

## Appendix

In this appendix, we will give some proofs of the main results.

### Proof of Theorem 2.1

#### Proof

Let $J(E_{0}),J(E_{1})$ denote the Jacobian matrix of system (1.2) at the equilibria $E_{0}$ and $E_{1}$, respectively, then we have

$$\begin{aligned} \mathbf{J}(X)= \begin{pmatrix} -(\beta _{1}-\frac{\beta _{2}I}{\eta +I}){I}-\mu & -(\beta _{1}- \frac{\beta _{2}I}{\eta +I}){S}+ \frac{\beta _{2}\eta {SI}}{(\eta +I)^{2}}-\mu (1-\alpha )q \\ (\beta _{1}-\frac{\beta _{2}I}{\eta +I}){I} & -(p\mu +\gamma )+ ( \beta _{1}-\frac{\beta _{2}I}{\eta +I}){S}- \frac{\beta _{2}\eta {IS}}{(\eta +I)^{2}} \end{pmatrix}, \end{aligned}$$

thus

A.1 $$\begin{aligned} \mathbf{J}(E_{0})={}& \begin{pmatrix} -\mu & -\beta _{1}{S_{0}}-\mu (1-\alpha )q \\ 0 & -(p\mu +\gamma )+\beta _{1}{S_{0}} \end{pmatrix} \\ ={}& \begin{pmatrix} -\mu & -\beta _{1}(1-\alpha )-\mu (1-\alpha )q \\ 0 & (p\mu +\gamma )(R_{0}-1) \end{pmatrix} \end{aligned}$$

and

A.2 $$\begin{aligned} \mathbf{J}(E_{1})={}& \begin{pmatrix} -(\beta _{1}-\frac{\beta _{2}I^{*}}{\eta +I^{*}}){I^{*}}-\mu & -( \beta _{1}-\frac{\beta _{2}I^{*}}{\eta +I^{*}}){S^{*}}+ \frac{\beta _{2}\eta {S^{*}I^{*}}}{(\eta +I^{*})^{2}}-\mu (1-\alpha )q \\ (\beta _{1}-\frac{\beta _{2}I^{*}}{\eta +I^{*}}){I^{*}} & -(p\mu + \gamma )+ (\beta _{1}-\frac{\beta _{2}I^{*}}{\eta +I^{*}}){S^{*}}- \frac{\beta _{2}\eta {I^{*}S^{*}}}{(\eta +I^{*})^{2}} \end{pmatrix} \\ ={}& \begin{pmatrix} -(\beta _{1}-\frac{\beta _{2}I^{*}}{\eta +I^{*}}){I^{*}}-\mu & -(p \mu +\gamma )+\frac{\beta _{2}\eta {S^{*}I^{*}}}{(\eta +I^{*})^{2}}- \mu (1-\alpha )q \\ (\beta _{1}-\frac{\beta _{2}I^{*}}{\eta +I^{*}}){I^{*}} & - \frac{\beta _{2}\eta {I^{*}S^{*}}}{(\eta +I^{*})^{2}} \end{pmatrix}. \end{aligned}$$

From (A.1), we can know that all characteristic values have negative real parts if and only if $R_{0}<1$, according to Routh–Hurwitz criteria, $E_{0}$ is locally stable if $R_{0}<1$.

For (A.2), we have

$$\begin{aligned} \operatorname{tr} \bigl(J(E_{1}) \bigr)={}&{-} \biggl(\beta _{1}-\frac{\beta _{2}I^{*}}{\eta +I^{*}} \biggr){I^{*}}- \mu -(p\mu +\gamma )+ \biggl(\beta _{1}-\frac{\beta _{2}I^{*}}{\eta +I^{*}} \biggr){S^{*}}- \frac{\beta _{2}\eta {I^{*}S^{*}}}{(\eta +I^{*})^{2}} \\ ={}&{- }\biggl(\beta _{1}-\frac{\beta _{2}I^{*}}{\eta +I^{*}} \biggr){I^{*}}-\mu - \frac{\beta _{2}\eta {I^{*}S^{*}}}{(\eta +I^{*})^{2}}< 0, \\ \det \bigl(J(E_{1}) \bigr)={}& \biggl(\beta _{1}- \frac{\beta _{2}I^{*}}{\eta +I^{*}} \biggr){I^{*}} \frac{\beta _{2}\eta {I^{*}S^{*}}}{(\eta +I^{*})^{2}}+ \frac{\mu \beta _{2}\eta {I^{*}S^{*}}}{(\eta +I^{*})^{2}}+(p\mu + \gamma ) \biggl(\beta _{1}- \frac{\beta _{2}I^{*}}{\eta +I^{*}} \biggr){I^{*}} \\ &{}- \biggl(\beta _{1}-\frac{\beta _{2}I^{*}}{\eta +I^{*}} \biggr){I^{*}} \frac{\beta _{2}\eta {S^{*}I^{*}}}{(\eta +I^{*})^{2}}+\mu (1-\alpha )q \biggl( \beta _{1}- \frac{\beta _{2}I^{*}}{\eta +I^{*}} \biggr){I^{*}} \\ ={}&\frac{\mu \beta _{2}\eta {I^{*}S^{*}}}{(\eta +I^{*})^{2}}+ \bigl[p\mu + \gamma +\mu (1-\alpha )q \bigr] \biggl(\beta _{1}- \frac{\beta _{2}I^{*}}{\eta +I^{*}} \biggr){I^{*}}>0. \end{aligned}$$

Thus, $E_{1}$ is locally asymptotically stable if $R_{0}>1$.

Choose a Dulac function $B=\frac{1}{SI}$. Denote

$$\begin{aligned} &F=- \biggl(\beta _{1}-\frac{\beta _{2}I}{\eta +I} \biggr){SI}-\mu {S}-(1-\alpha ) \mu {q}I+(1-\alpha ){\mu }, \\ &G= \biggl(\beta _{1}-\frac{\beta _{2}I}{\eta +I} \biggr){SI}-(p\mu +\gamma )I. \end{aligned}$$

Note that

$$\begin{aligned} &\frac{\partial {(BF)}}{\partial {S}}=-\frac{(1-\alpha ){\mu }}{S^{2}I}+ \frac{(1-\alpha )\mu {q}}{S^{2}}=- \frac{\mu (1-\alpha )(1-qI)}{S^{2}I}, \\ &\frac{\partial {(BG)}}{\partial {I}}=- \frac{\mu \beta _{2}}{(\eta +I)^{2}}, \\ &\frac{\partial {(BF)}}{\partial {S}}+\frac{\partial {(BG)}}{\partial {I}}=- \frac{\mu (1-\alpha )(1-qI)}{S^{2}I}- \frac{\mu \beta _{2}}{(\eta +I)^{2}}< 0, \end{aligned}$$

in the interior of the positive invariant set Γ. The Dulac criterion holds and there are no close orbits in Γ. Incorporating local stability of $E_{0}$ and $E_{1}$, this proves that $E_{0}$ is globally asymptotically stable if $R_{0} < 1$ and $E_{1}$ is globally asymptotically stable if $R_{0} > 1$. The proof is complete. □

### Proof of Theorem 3.1

#### Proof

Owing to system (1.3), we get

$$\begin{aligned} d \bigl(S (s )+I (s ) \bigr)={}& \bigl\{ -\mu S (s )- \bigl[ \mu (1- \alpha q)+\gamma \bigr]I (s )+\mu (1-\alpha ) \bigr\} \,ds \\ < {}& \bigl\{ -\mu S (s )-\mu (1-\alpha q)I (s )+\mu (1- \alpha ) \bigr\} \,ds \\ < {}& \bigl\{ -\mu (1-\alpha q) \bigl(S (s )+I (s ) \bigr)+\mu (1- \alpha ) \bigr\} \,ds, \end{aligned}$$

so, by integration we check

$$ S (s )+I (s )< \frac{1-\alpha }{1-\alpha q}+ \biggl[S(0)+I(0)- \frac{1-\alpha }{1-\alpha q} \biggr]e^{-\mu (1-\alpha q)s} \quad \text{for all }s \in [0,t]\text{ a.s.} $$

Then $S (s )+I (s )<\frac{1-\alpha }{1-\alpha q}<1$. In addition

$$\begin{aligned} d \bigl(S (s )+I (s ) \bigr)={}& \bigl\{ -\mu S (s )- \bigl[ \mu (1- \alpha q)+\gamma \bigr]I (s )+\mu (1-\alpha ) \bigr\} \,ds \\ >{}& \bigl\{ -\mu S (s )-[\mu +\gamma ]I (s )+\mu (1- \alpha ) \bigr\} \,ds \\ >{}& \bigl\{ -[\mu +\gamma ] \bigl(S (s )+I (s ) \bigr)+\mu (1- \alpha ) \bigr\} \,ds, \end{aligned}$$

so, by integration we check

$$ S (s )+I (s )> \frac{\mu (1-\alpha )}{\mu +\gamma }+ \biggl[S(0)+I(0)- \frac{\mu (1-\alpha )}{\mu +\gamma } \biggr]e^{-(\mu +\gamma )s} \quad \text{for all }s\in [0,t]\text{ a.s.} $$

Hence $S (s )+I (s )> \frac{\mu (1-\alpha )}{\mu +\gamma }$. So

A.3 $$ S (s ),I (s )\in \biggl( \frac{\mu (1-\alpha )}{\mu +\gamma },1 \biggr) \quad \text{for all }s \in [0,t]\quad \text{a.s.} $$

We can easily see that the coefficients of system (1.3) are locally Lipschitz continuous for any given initial value $(S(0),I(0))\in \mathbb{R}_{+}^{2}$. Hence, there is a unique local solution $(S(t),I(t))$ on $t\in [0,\tau _{e})$, where $\tau _{e}$ is the explosion time (see [41]). To show that this solution is global, we only need to prove that $\tau _{e}=\infty $ a.s. Let $k_{0}\geq 0$ be sufficiently large so that $(S(0),I(0))$ all lie within the interval $[\frac{1}{k_{0}},k_{0}]$. For each integer $k\geq {k_{0}}$, define the following stopping time:

$$ \tau _{k}=\inf \biggl\{ t\in [0,\tau _{e}): \min \bigl\{ \bigl(S (t ),I (t ) \bigr) \bigr\} \leq \frac{1}{k} \text{ or } \max \bigl\{ \bigl(S (t ),I (t ) \bigr) \bigr\} \geq {k} \biggr\} , $$

where throughout this paper, we set $\inf {\O }{=}\infty $ (and as usual Ø denotes the empty set). According to the definition, $\tau _{k}$ is increasing as $k\rightarrow \infty $. Set $\tau _{\infty }{=}\lim_{k\rightarrow \infty }\tau _{k}$, whence $\tau _{\infty }\leq \tau _{e}$ a.s. Namely, we need to show that $\tau _{\infty }{=}\infty $ a.s. We assumed that there exist a pair of constants $T>0$ and $\epsilon \in (0,1 )$ such that

$$ P\{\tau _{\infty }\leq {T}\}>\epsilon. $$

As a result, there is an integer $k_{1}\geq {k_{0}}$ such that

A.4 $$ P\{\tau _{k}\leq {T}\}>\epsilon \quad \text{for all }k\geq {k_{1}}. $$

Now define a $C^{2}$-function $V:\mathbb{R}_{+}^{2}\rightarrow \overline{\mathbb{R}}_{+}$, where $\overline{\mathbb{R}}_{+}=\{x\in \mathbb{R}:x\geq 0\}$, by

$$ V(t)=S-1-\log S+ I-1-\log I. $$

The nonnegativity of this function can be seen from $u-1-\log {u}\geq {0}$, $\forall {u}\geq 0$. Let $k\geq {k_{0}}$ and $T>{0}$ be arbitrary. Applying to It$\hat{o}^{,}s$ formula, we obtain

$$ dV(t)=LV(t)\,dt- \bigl[\sigma (S-1)I-\sigma (I-1)S \bigr]\,dB(t), $$

where

$$\begin{aligned} LV(t)={}& \biggl(1-\frac{1}{S} \biggr) \biggl(- \biggl(\beta _{1}- \frac{\beta _{2}I}{\eta +I} \biggr){SI}-\mu {S}-(1-\alpha )\mu {q}I+(1- \alpha ){ \mu } \biggr) \\ &{}+ \biggl(1-\frac{1}{I} \biggr) \biggl( \biggl(\beta _{1}- \frac{\beta _{2}I}{\eta +I} \biggr){SI}-(p\mu +\gamma )I \biggr)+ \frac{\sigma ^{2}S^{2}I^{2}}{2S^{2}}+ \frac{\sigma ^{2}S^{2}I^{2}}{2I^{2}} \\ ={}&(1-\alpha )\mu + \biggl(\beta _{1}-\frac{\beta _{2}I}{\eta +I} \biggr){I}+\mu +p \mu +\gamma +\frac{(1-\alpha )\mu {q}I}{S}+ \frac{\sigma ^{2}(S^{2}+I^{2})}{2} \\ &{}-\mu S- \bigl[(1-\alpha )\mu {q}+p\mu +\gamma \bigr]I- \biggl(\beta _{1}- \frac{\beta _{2}I}{\eta +I} \biggr){S} \\ \leq {}&(1-\alpha )\mu +\beta _{1} I+\mu +p\mu +\gamma + \frac{(1-\alpha )\mu {q}I}{S}+\frac{\sigma ^{2}(S^{2}+I^{2})}{2}+ \beta _{2}{S}, \end{aligned}$$

owing to (A.3), we have thus

$$ LV(t)\leq (1-\alpha )\mu +\beta _{1}+\mu +p\mu +\gamma +( \mu + \gamma )q+\sigma ^{2}+\beta _{2}\doteq B. $$

Then

A.5 $$ dV(t)=B\,dt- \bigl[\sigma (S-1)I-\sigma (I-1)S \bigr]\,dB(t) .$$

Integrating both sides (A.5) from 0 to $T\wedge {\tau _{k}}$ and taking expectations, then we can obtain

A.6 $$ \mathbb{E}V \bigl(S(T\wedge {\tau _{k}}),I(T\wedge {\tau _{k}}) \bigr) \leq {V \bigl(S (0 ),I (0 ) \bigr)}+BT< \infty. $$

Set $\Omega _{k}=\{\tau _{k}\leq {t}\}$ for $k\geq {k_{1}}$ by (A.4), $P (\Omega _{k} )\geq \epsilon $. Notice that, for every $\omega \in \Omega _{k}$, there is at least one of $S (\tau _{k},\omega )$, $I (\tau _{k},\omega )$ that equals either *k* or $\frac{1}{k}$. Hence $V(S (\tau _{k},\omega )$, $I (\tau _{k},\omega )$ is no less than

$$ k-1-\log {k} \quad\text{or}\quad \frac{1}{k}-1-\log {\frac{1}{k}}= \frac{1}{k}-1+\log {k}. $$

Consequently,

A.7 $$ V \bigl(S(\tau _{k},\omega ),I(\tau _{k},\omega ) \bigr)\geq {(k-1- \log {k})}\wedge \biggl(\frac{1}{k}-1+\log {k} \biggr), $$

where $a\wedge {b}$ denotes the minimum of *a* and *b*. In view of (A.6) and (A.7), we have

$$\begin{aligned} V \bigl(S (0 ),I (0 ) \bigr)+BT&\geq { \mathbb{E}} \bigl[I_{\Omega _{k}}V \bigl(S(\tau _{k},\omega ),I(\tau _{k}, \omega ) \bigr) \bigr] \\ &\geq \epsilon \biggl[(k-1-\log {k})\wedge \biggl(\frac{1}{k}-1+\log {k} \biggr) \biggr], \end{aligned}$$

where $I_{\Omega _{k}}$ is the indicator function of $\Omega _{k}$. Let $k\rightarrow \infty $ leads to the contradiction

$$ \infty >V \bigl(S (0 ),I (0 ) \bigr)+BT= \infty. $$

Therefore, we must have $\tau _{\infty }=\infty $ a.s. □

### Proof of Theorem 3.2

#### Proof

Making use of It$\hat{o}^{,}s$ formula for ln*I*, we have

A.8 $$\begin{aligned} d\ln I&= \biggl[ \biggl(\beta _{1}-\frac{\beta _{2}I}{\eta +I} \biggr){S}-(p\mu +\gamma )- \frac{\sigma ^{2}S^{2}}{2}) \biggr]\,dt+\sigma S\,dB(t) \\ &\leq \biggl[\beta _{1} S-(p\mu +\gamma )-\frac{\sigma ^{2}S^{2}}{2} \biggr] \,dt+ \sigma S\,dB(t) \\ &= \biggl\{ - \biggl[\frac{\sigma S}{\sqrt{2}}- \frac{\sqrt{2}\beta _{1}}{2\sigma } \biggr]^{2}+ \frac{\beta ^{2}_{1}}{2\sigma ^{2}}-(p\mu +\gamma ) \biggr\} \,dt+ \sigma S \,dB(t) \\ &\leq \biggl\{ \frac{\beta _{1}^{2}}{2\sigma ^{2}}-(p\mu +\gamma ) \biggr\} \,dt+\sigma S\,dB(t). \end{aligned}$$

Integrating Eq. (A.8) from 0 to *t* and dividing by *t* on both sides, we have

A.9 $$ \frac{\ln I(t)-\ln I(0)}{t}\leq \frac{\beta _{1}^{2}}{2\sigma ^{2}}-(p \mu +\gamma )+ \frac{M(t)}{t}, $$

where $M(t)=\int _{0}^{t}\sigma S \,dB(t)$ is a real-value continuous local martingale, since we have the quadratic variations, we can have

$$ \limsup_{t\rightarrow \infty }\langle {M,M\rangle }_{t} \leq \sigma ^{2}< \infty. $$

By the large number theorem for the martingale (see [41]), we can get

A.10 $$ \lim_{t\rightarrow \infty }\frac{M(t)}{t}=0,\quad \text{a.s.} $$

According to (A.9) and (A.10), we have

A.11 $$ \limsup_{t\rightarrow \infty }\frac{\ln I(t)}{t}\leq \frac{\beta _{1}^{2}}{2\sigma ^{2}}-(p\mu +\gamma ), \quad \text{a.s.} $$

That is to say, if $\sigma ^{2}>\frac{\beta _{1}^{2}}{2(p\mu +\gamma )}$, we obtain

$$ \lim_{t\rightarrow \infty }I(t)=0, \quad \text{a.s.} $$

On the other hand, let $x= S,x\in (0,1],f(x)=\beta _{1} x -\frac{\sigma ^{2}x^{2}}{2}=-( \frac{\sigma x}{\sqrt{2}}-\frac{\beta _{1}\sqrt{2}}{2\sigma })^{2}$, if $\frac{\sigma }{\sqrt{2}}\leq \frac{\beta _{1}\sqrt{2}}{2\sigma }$, that is, $\sigma ^{2}\leq \beta _{1}$, $f(x)$ has the max value $f(1)=\beta _{1}-\frac{\sigma ^{2}}{2}$, namely, $S=1$ by Eq. (A.8), we have

A.12 $$\begin{aligned} d\ln I&= \biggl[ \biggl(\beta _{1}-\frac{\beta _{2}I}{\eta +I} \biggr){S}-(p\mu +\gamma )- \frac{\sigma ^{2}S^{2}}{2}) \biggr]\,dt+\sigma S\,dB(t) \\ &\leq \biggl[\beta _{1} S-(p\mu +\gamma )-\frac{\sigma ^{2}S^{2}}{2} \biggr] \,dt+ \sigma S\,dB(t) \\ &= \biggl\{ - \biggl[\frac{\sigma S}{\sqrt{2}}- \frac{\sqrt{2}\beta _{1}}{2\sigma } \biggr]^{2}+ \frac{\beta ^{2}_{1}}{2\sigma ^{2}}-(p\mu +\gamma ) \biggr\} \,dt+ \sigma S \,dB(t) \\ &\leq \biggl\{ \beta _{1}-\frac{\sigma ^{2}}{2}-(p\mu +\gamma ) \biggr\} \,dt+\sigma S\,dB(t). \end{aligned}$$

Integrating Eq. (A.12) from 0 to *t* and dividing by *t* on both sides, we have

A.13 $$ \frac{\ln I(t)-\ln I(0)}{t}\leq \beta _{1}-\frac{\sigma ^{2}}{2}-(p \mu +\gamma )+\frac{M(t)}{t}, $$

then

A.14 $$ \limsup_{t\rightarrow \infty }\frac{\ln I(t)}{t}\leq \beta _{1}- \frac{\sigma ^{2}}{2}-(p\mu +\gamma ),\quad \text{a.s.} $$

That is to say, if $\sigma ^{2}\leq \beta _{1}$ and $\beta _{1}<\frac{\sigma ^{2}}{2}+(p\mu +\gamma )$, we have

$$ \limsup_{t\rightarrow \infty }\frac{\ln I(t)}{t}\leq \beta _{1}- \frac{\sigma ^{2}}{2}-(p\mu +\gamma )< 0, \quad \text{a.s.} $$

Hence, we can get

$$ \lim_{t\rightarrow \infty }I(t)=0,\quad \text{a.s.} $$

Furthermore, we have

A.15 $$ \lim_{t\rightarrow \infty } \bigl\langle I(t) \bigr\rangle =0, \quad\text{a.s.} $$

For the system (1.3), we have

A.16 $$ d(S+I)= \bigl\{ (1-\alpha )\mu -\mu S- \bigl[\mu (1-\alpha q)+\gamma \bigr]I \bigr\} \,dt. $$

Integrating Eq. (A.16) from 0 to *t* and dividing by *t* on both sides, we have

A.17 $$ \frac{S(t)-S(0)+I(t)-I(0)}{t}=(1-\alpha )\mu -\mu \bigl\langle S(t) \bigr\rangle - \bigl[\mu (1-\alpha q)+\gamma \bigr] \bigl\langle I(t) \bigr\rangle . $$

Then

A.18 $$ \bigl\langle S(t) \bigr\rangle =(1-\alpha )- \frac{[\mu (1-\alpha q)+\gamma ]}{\mu } \bigl\langle I(t) \bigr\rangle +\Psi (t), $$

where

$$ \Psi (t)=\frac{1}{\mu t} \bigl[S(0)+I(0)-S(t)-I(t) \bigr], $$

we obtain

A.19 $$ \lim_{t\rightarrow \infty }\Psi (t)=0. $$

Combining (A.15) and (A.19), we have

$$ \lim_{t\rightarrow \infty } \bigl\langle S(t) \bigr\rangle =1- \alpha,\quad \text{a.s.} $$

This completes the proof. □

### Proof of Theorem 3.3

#### Proof

Making use of Itô’s formula, we obtain

A.20 $$\begin{aligned} d\ln I&= \biggl[ \biggl(\beta _{1}-\frac{\beta _{2}I}{\eta +I} \biggr){S}-(p\mu + \gamma )-\frac{\sigma ^{2}S^{2}}{2} \biggr]\,dt+\sigma S\,dB(t) \\ &\leq \biggl[\beta _{1} S-(p\mu +\gamma )-\frac{\sigma ^{2}S^{2}}{2} \biggr] \,dt+\sigma S\,dB(t). \end{aligned}$$

Integrating Eq. (A.20) from 0 to *t* and dividing by *t* on both sides, we have

A.21 $$\begin{aligned} \frac{\ln I(t)-\ln I(0)}{t}&\leq \beta _{1} \bigl\langle S(t) \bigr\rangle -(p \mu +\gamma )-\frac{\sigma ^{2}}{2} \bigl\langle S^{2}(t) \bigr\rangle + \frac{M(t)}{t} \\ &\leq \beta _{1} \bigl\langle S(t) \bigr\rangle -(p\mu +\gamma )- \frac{\sigma ^{2}}{2} \bigl\langle S(t) \bigr\rangle ^{2}+ \frac{M(t)}{t}. \end{aligned}$$

In view of (A.18), we have

$$ -\frac{\sigma ^{2}}{2} \bigl\langle S(t) \bigr\rangle ^{2}=- \frac{\sigma ^{2}}{2} \biggl[(1-\alpha )+\Psi (t)-\frac{\mu (1-\alpha q)+\gamma }{\mu } \bigl\langle I(t) \bigr\rangle \biggr]^{2}. $$

So

A.22 $$\begin{aligned} &\frac{\ln I(t)-\ln I(0)}{t} \\ &\quad\leq \beta _{1} \bigl\langle S(t) \bigr\rangle -(p \mu +\gamma )-\frac{\sigma ^{2}}{2} \bigl\langle S(t) \bigr\rangle ^{2}+ \frac{M(t)}{t} \\ &\quad=\beta _{1} \biggl\{ (1-\alpha )-\frac{\mu (1-\alpha q)+\gamma }{\mu } \bigl\langle I(t) \bigr\rangle +\Psi (t) \biggr\} -(p\mu +\gamma ) \\ &\qquad{}-\frac{\sigma ^{2}}{2} \biggl[(1-\alpha )+\Psi (t)- \frac{\mu (1-\alpha q)+\gamma }{\mu } \bigl\langle I(t) \bigr\rangle \biggr]^{2}+ \frac{M(t)}{t} \\ &\quad=\beta _{1}(1-\alpha )-\frac{\beta _{1}\mu (1-\alpha q)+\gamma }{\mu } \bigl\langle I(t) \bigr\rangle +\beta _{1}\Psi (t)-(p\mu +\gamma ) \\ &\qquad{}-\frac{\sigma ^{2}}{2}(1-\alpha )^{2}-\frac{\sigma ^{2}}{2}\Psi ^{2}(t)- \sigma ^{2}(1-\alpha )\Psi (t)+\frac{M(t)}{t} \\ &\qquad{}+ \frac{\sigma ^{2}[(1-\alpha )+\Psi (t)][\mu (1-\alpha q)+\gamma ]}{\mu } \bigl\langle I(t) \bigr\rangle - \frac{\sigma ^{2}[\mu (1-\alpha q)+\gamma ]^{2}}{2\mu ^{2}} \bigl\langle I(t) \bigr\rangle ^{2} \\ &\quad\leq \beta _{1}(1-\alpha )-(p\mu +\gamma )-\frac{\sigma ^{2}}{2}(1- \alpha )^{2}+\beta _{1}\Psi (t)+\frac{M(t)}{t} \\ &\qquad{}+ \frac{\sigma ^{2}[(1-\alpha )+\Psi (t)][\mu (1-\alpha q)+\gamma ]}{\mu } \bigl\langle I(t) \bigr\rangle -\frac{\beta _{1}\mu (1-\alpha q)+\gamma }{\mu } \bigl\langle I(t) \bigr\rangle . \end{aligned}$$

Hence

A.23 $$\begin{aligned} \bigl\langle I(t) \bigr\rangle \leq {}&\frac{\mu [\beta _{1}(1-\alpha )-(p\mu +\gamma +\frac{\sigma ^{2}}{2}(1-\alpha )^{2})]}{[\mu (1-\alpha q)+\gamma ] (\beta _{1}-\sigma ^{2}[(1-\alpha )+\Psi (t)])} \\ &{}+\frac{\mu }{[\mu (1-\alpha q)+\gamma ](\beta _{1}-\sigma ^{2}[(1-\alpha )+\Psi (t)])} \biggl[\beta _{1}\Psi (t)+\frac{M(t)}{t} \biggr]. \end{aligned}$$

In view of (A.10) and (A.19), we have

A.24 $$ \limsup_{t\rightarrow \infty } \bigl\langle I(t) \bigr\rangle \leq \frac{\mu [\beta _{1}(1-\alpha )-(p\mu +\gamma +\frac{\sigma ^{2}}{2}(1-\alpha )^{2})]}{ [\mu (1-\alpha q)+\gamma ][\beta _{1}-\sigma ^{2}(1-\alpha )]}=I_{1},\quad \text{a.s.} $$

On the other hand,

A.25 $$\begin{aligned} d\ln I&= \biggl[ \biggl(\beta _{1}-\frac{\beta _{2}I}{\eta +I} \biggr){S}-(p\mu + \gamma )-\frac{\sigma ^{2}S^{2}}{2} \biggr]\,dt+\sigma S\,dB(t) \\ &\geq \biggl[(\beta _{1}-\beta _{2}) S-(p\mu +\gamma )- \frac{\sigma ^{2}S^{2}}{2} \biggr]\,dt+\sigma S\,dB(t). \end{aligned}$$

So,

A.26 $$\begin{aligned} &\frac{\ln I(t)-\ln I(0)}{t} \\ &\quad\geq(\beta _{1}-\beta _{2}) \bigl\langle S(t) \bigr\rangle -\frac{\sigma ^{2}}{2} \bigl\langle S^{2}(t) \bigr\rangle +\frac{M(t)}{t} \\ &\quad\geq (\beta _{1}-\beta _{2}) \biggl[(1-\alpha )- \frac{\mu (1-\alpha q)+\gamma }{\mu } \bigl\langle I(t) \bigr\rangle +\Psi (t) \biggr]- \frac{\sigma ^{2}}{2}+\frac{M(t)}{t}. \end{aligned}$$

Hence,

A.27 $$ \bigl\langle I(t) \bigr\rangle \geq \frac{\mu }{(\beta _{1}-\beta _{2})[\mu (1-\alpha q)+\gamma ]} \biggl[ ( \beta _{1}-\beta _{2}) \bigl[(1-\alpha )+\Psi (t) \bigr]- \frac{\sigma ^{2}}{2}+\frac{M(t)}{t} \biggr]. $$

In the light of (A.10) and (A.19), we have

A.28 $$ \liminf_{t\rightarrow \infty } \bigl\langle I(t) \bigr\rangle \geq \frac{\mu ((\beta _{1}-\beta _{2})(1-\alpha )-\sigma ^{2})}{2 (\beta _{1}-\beta _{2})[\mu (1-\alpha q)+\gamma ]}=I_{2}\quad \text{a.s.} $$

Thus from (A.24) and (A.28), we have

$$ I_{2}\leq \liminf_{t\rightarrow \infty } \bigl\langle I(t) \bigr\rangle \leq \limsup_{t\rightarrow \infty } \bigl\langle I(t) \bigr\rangle \leq I_{1}\quad \text{a.s.} $$

 □
